# Function of Green Tea Catechins in the Brain: Epigallocatechin Gallate and its Metabolites

**DOI:** 10.3390/ijms20153630

**Published:** 2019-07-25

**Authors:** Monira Pervin, Keiko Unno, Akiko Takagaki, Mamoru Isemura, Yoriyuki Nakamura

**Affiliations:** 1 Tea Science Center, Graduate School of Integrated Pharmaceutical and Nutritional Sciences, University of Shizuoka, Shizuoka 422-8526, Japan; 2 R&D group, Mitsui Norin Co. Ltd., Shizuoka 426-0133, Japan

**Keywords:** blood–brain barrier, catechin, cognition, epigallocatechin gallate, green tea, microbiota, 5-(3,5-dihydroxyphenyl)-γ-valerolactone

## Abstract

Over the last three decades, green tea has been studied for its beneficial effects, including anti-cancer, anti-obesity, anti-diabetes, anti-inflammatory, and neuroprotective effects. At present, a number of studies that have employed animal, human and cell cultures support the potential neuroprotective effects of green tea catechins against neurological disorders. However, the concentration of (−)-epigallocatechin gallate (EGCG) in systemic circulation is very low and EGCG disappears within several hours. EGCG undergoes microbial degradation in the small intestine and later in the large intestine, resulting in the formation of various microbial ring-fission metabolites which are detectable in the plasma and urine as free and conjugated forms. Recently, in vitro experiments suggested that EGCG and its metabolites could reach the brain parenchyma through the blood–brain barrier and induce neuritogenesis. These results suggest that metabolites of EGCG may play an important role, alongside the beneficial activities of EGCG, in reducing neurodegenerative diseases. In this review, we discuss the function of EGCG and its microbial ring-fission metabolites in the brain in suppressing brain dysfunction. Other possible actions of EGCG metabolites will also be discussed.

## 1. Introduction

Tea is derived from the leaves and buds of the plant *Camellia sinensis* L. (Theaceae). Among the different types of tea, such as green tea, black tea, and oolong tea, the health benefits of green tea have been most extensively studied [1,2]. These include anti-cancer [3,4], anti-obesity [5,6,7], anti-diabetes [8,9], and neuroprotective effects [10,11,12]. The antioxidant and metal chelating [13,14], anti-carcinogenic [15], anti-apoptotic [16,17], pro-apoptotic, and anti-inflammatory [14,18] properties of catechins are greatly associated with their beneficial health effects, including suppressing neurodegenerative diseases.

**Figure 1:**
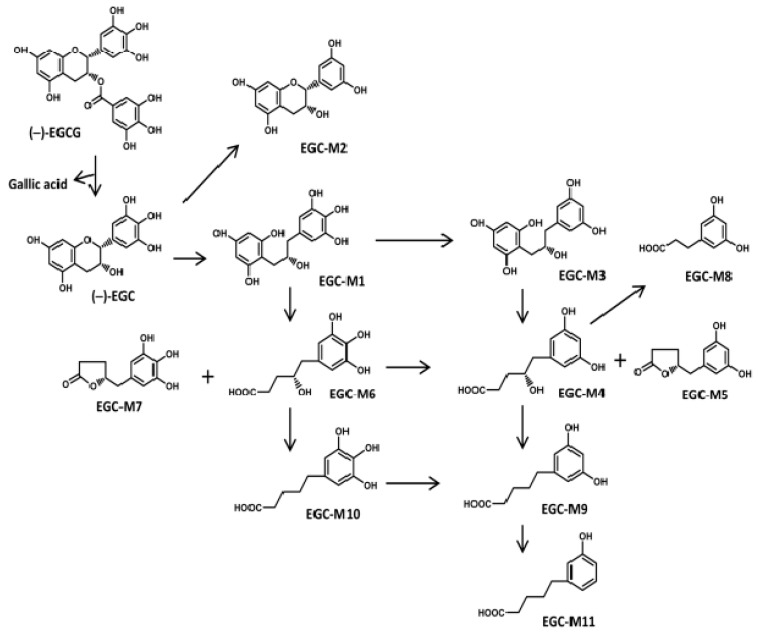
Chemical structures of EGCG metabolites based on data from Takagaki et al. [41].

Compared to other beverages, green tea is rich in catechins. According to Khokhar et al., 100 mL of green tea (1 g of dry tea leaves brewed for 5 min in 100 mL of hot water) contains on average 67 ± 11 mg of total catechins, including about 30 mg of (−)-epigallocatechin gallate (EGCG), whereas black tea contains 15.4 mg of catechins [19]. In green tea catechins, the main active molecule, EGCG (Figure 1), an ester of (−)-epigallocatechin (EGC) and gallic acid (GA), represents 50–80% of the total catechin content, followed by EGC, (−)-epicatechin gallate (ECG), (−)-epicatechin (EC), and (+)-catechin (C) [20]. Numerous beneficial effects of EGCG have been reported on cognitive function and oxidative damage [21,22,23,24]. Several epidemiological studies also showed the association between drinking tea and the beneficial effects on cognitive function [25,26,27,28]. For example, a cross-sectional study by Kuriyama et al. showed that daily ingestion of one or two cups of green tea significantly reduced cognitive impairment [25]. In another clinical study by Ide et al., the consumption of green tea (2 g/day) for 3 months significantly improved cognitive function and also reduced the progression of cognitive dysfunction [29].

Male Wistar rats that orally ingested EGCG showed a peak concentration at 1–2 h in systemic circulation, and it remained present in trace amounts after 4 h [30]. Much of orally ingested EGCG undergoes intestinal microbial degradation in the small intestine to EGC and GA, and later in the large intestine, resulting in the formation of various colonic microbial ring-fission metabolites, which are detectable in the plasma and urine [31,32,33,34]. These metabolites can exhibit biological activities, and some of them may be attributed to the action of EGCG.

This review discusses the function of EGCG and its metabolites as well as their possible action in the brain in suppressing brain dysfunction. In addition, recent data of other functions of EGCG metabolites are described.

## 2. Bioactivity of EGCG and Its Metabolites in the Brain

### 2.1. Absorption and Bioavailability of EGCG

EGCG is poorly absorbed by the body, it reaches the blood circulation at a very low micromolar concentration, and then it disappears from plasma within several hours [30,35,36,37,38]. The oral bioavailability of EGCG is estimated to be about 0.1 to 0.3% in rats and humans [25,26,30,35].

#### Catechin Ring-Fission Products

EGCG was found to be hydrolyzed by intestinal microbiota to produce EGC and GA. EGC was further degraded to some kinds of ring-fission metabolites in the gut tract. In the large intestine, there are 11 colonic microbial ring-fission metabolites of EGC (EGC-M1–M11) (Table 1, Figure 1) as described by Takagaki et al., i.e. 1-(3,4,5-trihydroxyphenyl) 3-(2,4,6-trihydroxyphenyl)-propan-2-ol (EGC-M1), 4-dehydroxylated epigallocatechin (EGC-M2), 1-(3,5-dihydroxyphenyl)-3-(2,4,6-trihydroxyphenyl)-propan-2-ol (EGC-M3), 4-hydroxy-5-(3,5-dihydroxyphenyl) valeric acid (EGC-M4), 5-(3,5-dihydroxyphenyl)-γ-valerolactone (EGC-M5), 4-hydroxy-5-(3,4,5-trihydroxyphenyl) valeric acid (EGC-M6), 5-(3,4,5-trihydroxyphenyl)-γ-valerolactone (EGC-M7), 3-(3,5-dihydroxyphenyl) propionic acid (EGC-M8), 5-(3,5-dihydroxyphenyl) valeric acid (EGC-M9), 5-(3,4,5-trihydroxyphenyl) valeric acid (EGC-M10), and 5-(3-hydroxyphenyl) valeric acid (EGC-M11) [39,40,41]. Among them, EGC-M5 and EGC-M7 were found to be the main metabolites in mice, rat, and human plasma, urine, and bile [42].

The intestinal microbial ring-fission metabolites of EGCG are present in plasma as free and conjugated forms [31], and in vitro data suggested that they could reach the brain parenchyma through the blood–brain barrier (BBB) and induce neuritogenesis [43], suggesting that they might be important in suppressing neurodegenerative diseases.

The bioavailability of a compound or its metabolites can be determined by quantifying the concentration at the systematic blood flow and at the target organ [44]. It is very important to know the metabolic process and bioavailability of green tea catechins to evaluate their biological activity as well as to understand their beneficial effects on human health. EGCG has much lower bioavailability than other components in catechins [36,45]. For example, after intragastric administration of decaffeinated green tea (200 mg/kg) to male Sprague–Dawley rats, 13.7% of EGC, 31.2% of EC, and 0.1% of EGCG appeared in the blood [36]. The bioavailability of EGCG is significantly different depending on the route of administration, such as intravenous, intragastric, or through peroral ingestion, since intravenously ingested EGCG can equally reach all tissues in a free state (without conjugate) compared to intragastric and peroral administration as a result of the high levels of EGCG in intravenous ingestion. It is much easier for tissues to absorb free EGCG (without conjugate) in intravenous ingestion compared to other routes of administration [38]. On the other hand, the absorption rate of EGCG in plasma was much better in peroral administration [46] compared to intragastric intubation, although the detailed mechanism is not clear [36]. Mice and rats show a difference in bioavailability. For example, in the mice model, there is higher absorption of EGCG (26.5%) [38] than in the rat model (1.6%) [36].

Aglycons (without sugar residues) from plant polyphenols are easily absorbed in the small intestine [47]. However, the majority of polyphenols in plants exist as a form of glycosides, esters, or polymers, and they cannot be absorbed directly from the intestine. Therefore, they are hydrolyzed by intestinal enzymes or gut microbiota. EGCG, the ester of epigallocatechin and GA, is metabolized by intestinal microbiota in rats [39,40,48,49].

In mice, the bioavailability of a single dose of pure EGCG was first reported by Lambert et al. The authors found that after intravenous (21.8 µmol/kg) and intragastric (163.8 µmol/kg) administration of EGCG to male CF-1 mice, the plasma levels of total EGCG reached about 2.7 ± 0.7 and 0.28 ± 0.08 µM, respectively. The levels of free EGCG in the liver, lung, small intestine, and colon were about 3.56, 2.66, 2.40, and 1.20 nmol/g, respectively. The levels of total EGCG in the small intestine and colon were 45.2 and 7.9 nmol/g, but the levels in the liver and lung could not be determined as the concentration was too low [38]. On the other hand, in male Sprague–Dawley rats, the plasma bioavailability of EGCG was 0.1~1.6%, suggesting that the rate of absorption in mice is much higher than in rats [36].

After [4^−3^H]EGCG (4 mg, 7.4 MBq/kg) was administered to male Wistar rats by intragastric gavage, the absorption, distribution, and excretion in blood, tissues, urine, and feces of EGCG and its metabolites were determined by tracing radioactivity using high-performance liquid chromatography (HPLC) analysis [31]. The results show that the radioactivity of EGCG mostly disappeared in the stomach by 72 h. Peak radioactivity in the small intestine, cecum, and large intestine was detected at 4 h (40.5% of the dose), 8 h (46.4% of the dose), and 8 h (13.2% of the dose), respectively, and the radioactivity was markedly reduced by 24 h and had almost disappeared by 72 h in these tissues. The level of radioactivity in the blood was low at 4 h, began to increase after 8 h, peaked at 24 h, and thereafter decreased. The urinary levels of two major radioactive metabolites, 5-(5-hydroxyphenyl)-γ-valerolactone 3-*O*-β-glucuronide and EGC-M5 were 68% and 16.8% of the ingested radioactivity after 48 h. The authors suggested that intragastrically ingested EGCG is absorbed in the intestine within several hours (<8 h), and thereafter the EGCG metabolites and conjugates are absorbed from the large intestine (>8~48 h), distributed to various tissues via blood circulation, and finally excreted via urine [31]. The degradation of EGCG by gut microbiota could be an important factor in decreasing its bioavailability [50]. When male C57BL/6J mice were given water containing (per mL) ampicillin (1 mg), sulfamethoxazole (1.6 mg), and trimethoprim (0.32 mg) for 11 days and then given a 0.32% Polyphenon E diet containing 643 mg EGCG, 29 mg EGC, 74 mg ECG, 90 mg EC, 45 mg gallocatechin gallate, and 6 mg caffeine per g of Polyphenon E, the levels of EGCG in blood, liver, and urine increased. On the other hand, antibiotic treatment decreased the urinary levels of EGC-M7, the ring-fission metabolites of EGCG, and 5-(3,4-dihydroxyphenyl)-γ-valerolactone, a ring-fission metabolite of EC. This finding suggests that antibiotic treatment eliminated catechin-degrading microbiota in the gut and therefore, increased the levels of EGCG as well as decreased the ring-fission metabolites due to the presence of a low content of microbiota in the gut [50].

In male Sprague–Dawley rats that were given EGCG orally at 150 mg/kg, the plasma and the tissue distribution of EGCG were detected by developed HPLC with electrochemical detection [46]. After 2 h and 5 h of administration of EGCG, the levels of free (without conjugated) and total EGCG (with glucuronides, sulfates, and glucuronides/sulfates) in rat plasma were 0.7, 0.28, 0.82, and 0.5 µM, respectively. The authors also reported unpublished data showing that the plasma level of EGCG in rats 24 h after administration is 0.05 µM, suggesting that the EGCG level was markedly reduced 24 h after administration. The tissue levels of free EGCG in the small intestine and colon were 21.15 and 10.75, as well as 4.75 and 24.41 nmol/g at 2 and 5 h, respectively. They showed that the levels of free EGCG in the kidney, liver, spleen, lung, and brain were 1.02 and 0.54, 1.02 and 0.54, 0.1 and 0.12, 0.4 and 0.14, and 0.19 and 0.18 nmol/g at 2 and 5 h, respectively. These results indicate that the levels of EGCG in plasma and other tissues were high at 2 h and began to decrease 5 h after administration. Moreover, the plasma level of EGCG was very low 24 h after ingestion [46].

A human study by Warden et al. showed that after drinking black tea containing 16.74 mg of EGCG, 15.48 mg of EGC, 36.54 mg of EC, and 31.14 mg of ECG, the plasma concentration of EGCG was at the peak level between 5 and 8 h, but returned to baseline levels by 24 h. After tea ingestion over 6 h, the ingested catechins detected in plasma, urine, and feces were about 0.16%, 1.1%, and 0.42%, respectively, suggesting that level of absorption of catechins in humans is also quite low [51].

Microflora-mediated ring fission metabolites have also been identified in humans. EGCG was found to be hydrolyzed in the small intestine by intestinal microflora to produce EGC and GA and further degraded in the large intestine to produce various kinds of microbial ring fission metabolites [34,52,53]. In a human urinary metabolite profile, the ring-fission metabolites of tea catechins, such as 5-(3, 4-dihydroxyphenyl)-γ-valerolactone, EGC-M5, EGC-M7, and their glucuronide and sulfate conjugates, were found to be the major urinary metabolites at 12–24 h after ingestion of tea (200 mL of reconstituted green tea (from 3 g of tea solids)) in healthy male volunteers [34]. Two catechin ring-fission metabolites, EGC-M7 and 5-(3,4-dihydroxyphenyl)-γ-valerolactone, appeared in urine (4–8 µM) and in plasma (0.1–0.2 µM) approximately 13 h after ingestion of 20 mg/kg of decaffeinated green tea [53]. In addition, the cumulative urinary excretion of these microbial ring-fission metabolites was as high as 8–25 times the levels of ECG and EC [53]. A recent study on colonic ring-fission metabolism in humans identified various urinary metabolites derived from green tea flavan-3-ol (639 µmol of monomeric catechin and 88 µmol of oligomeric catechin), including EGC-M5, EGC-M7, 5-(4,5-dihydroxyphenyl)-γ-valerolactone, and 5-(hydroxyphenyl)-γ-valerolactone, with their glucuronide and sulphate conjugates [54]. The excretion rates of these ring-fission metabolites were as follows: EGC-M5-disulphate (163 µmol), EGC-M5-glucuronide (34.4 µmol), EGC-M7-sulphate (27.7 µmol), EGC-M7-glucuronide (12.1 µmol), methyl-EGC-M7-sulphate (54.7 µmol), methyl-EGC-M7-glucuronide (2.7 µmol), 5-(4,5-dihydroxyphenyl)-γ-valerolactone-disulphate (87.6 µmol), 5-(4,5-dihydroxyphenyl)-γ-valerolactone-glucuronide (16.8 µmol), 5-(hydroxyphenyl)-γ-valerolactone-sulphate (19.7 µmol), and 5-(hydroxyphenyl)-γ-valerolactone-glucuronide (6.6 µmol) [54]. In this study, the bioavailability of green tea flavan-3-ols was about 62% (the ratio between total metabolic excretion and total intake of flavan-3-ols) in 48 h which is higher than that reported previously (39%) in 24 h [52]. This study examined a more complete 48 h metabolic excretion profile and quantified a wider range of colonic microbial metabolites [54].

### 2.2. Blood–Brain Barrier Permeability of EGCG and Its Metabolites

The BBB is a dynamic system that separates circulating peripheral blood from brain neural tissue in the central nervous system. It is composed of endothelial cells connected through gap junctional proteins, astrocytes, pericytes, and extracellular matrix and works together to regulate the movement of ions, molecules, and cells between the blood and the brain to create a unique microenvironment for proper neuronal function [55]. Therefore, the BBB plays a significant role in transporting intravascular substances into the brain.

After male Sprague–Dawley rats were administrated EGCG at 50 mg/kg, the concentration of EGCG in various brain regions was measured by liquid chromatography tandem mass spectrometry (LC-MS/MS) [56]. The concentration of EGCG in various brain regions was about 5 ng/mL (0.01 µM) and ~4.95% of the orally administered EGCG (100 mg/kg) reached the systemic circulation. However, it was unclear whether EGCG was transferred from blood vessels into the parenchyma [56]. The concentration of EGCG in rat brain tissue (extracted consecutively with ethyl acetate and methanol) was determined to be about 0.5 nmol/g by chemiluminescence-detection HPLC (CL-HPLC) at 60 min after oral administration (500 mg/kg) in male Sprague–Dawley rats [57].

When the blood-to brain distribution ratios of C and EC which were administered (20 mg/kg) to male Sprague–Dawley rats via the femoral vein, which was measured by microdialysis sampling coupled with CL-HPLC, the ratios of C and EC were 0.0726 ± 0.0376 and 0.1065 ± 0.0531, respectively, as determined using the area under the curve for brain and blood [58]. In another study, the transport efficiency of C and EC at 30 mM was determined using two BBB cell lines, RBE-4 (rat brain endothelial cell) and hCMEC/D3 (human brain endothelial cell). Results showed that both C and EC effectively crossed the barrier in a time-dependent manner, and that the percentage of transport efficiency (% in 1 h) of EC (15.4 ± 0.6) was significantly higher than C (7.4 ± 0.7) [59].

Recently, we determined in vitro BBB permeability of EGCG and its metabolites (Table 2) by LC–MS/MS using a BBB kit (RBT-24, PharmaCo-Cell, Nagasaki, Japan) consisting of co-cultures of endothelial cells, pericytes, and astrocytes [43,60]. The in vitro BBB permeability (%, in 0.5 h) of EGCG, EGC, and GA was 4.00 ± 0.17, 4.96 ± 0.55, and 9.42 ± 1.01, respectively (the data from [43] are modified). GA exhibited a higher permeability than EGCG and EGC, perhaps due to the smaller molecular size of GA (MW 170.12) compared to EGCG (MW 458.372) and EGC (MW 306.27). The BBB permeability of EGC was lower than that of EC, and between EC and C. Lower BBB permeability of EGC than that of EC may be due to one more hydroxyl bond of EGC than EC, which affects its permeability. On the other hand, BBB permeability may be influenced by the presence of hydrophobicity of the galloyl bond [43,59,60].

The BBB permeability (%, in 0.5 h) of microbial ring-fission metabolites EGC-M5, and its conjugates, such as glucuronide of EGC-M5 (EGC-M5-GlcUA) and sulfate of EGC-M5 (EGC-M5-Sul), were 5.34 ± 0.23, 3.72 ± 0.01, and 4.34 ± 0.40, respectively. EGC-M5, with a smaller molecular size (MW 208.07), exhibited a slightly higher permeability than its conjugates EGC-M5-GlcUA (MW 384.11) and EGC-M5-Sul (MW 287.02), suggesting that the smaller molecular size of EGC-M5 caused its higher permeability [43].

### 2.3. Neuritogenic Activity of EGCG and Its Microbial Ring-Fission Metabolites

Since EGCG and its microbial ring-fission metabolites were able to reach brain parenchyma through the BBB, findings on how these bioactive compounds work in the brain and verification of their neuritogenic activity were needed. Human neuroblastoma SH-SY5Y cells (ATCC, CRL-2266) were used to assess neuritogenic activity as they are often used as in vitro models of neuronal function and differentiation [61]. In brief, SH-SY5Y cells were plated as 2.5 × 10^4^ cells/mL in a 24-well plate (500 μL of cell suspension/well). EGCG and its metabolites, which were dissolved in 0.01% DMSO, were added to the culture medium to make a final concentration of 0.01–1.0 μM, and cultured for ~72 h. Neurite length was measured by ImageJ software (Ver. 1.50i) [43,60]. Neurite length was significantly prolonged in cells treated with EGCG and EGC-M5 at 0.05 μM compared to control cells. In addition, SH-SY5Y cell growth was significantly enhanced by 0.05 μM EGCG and its metabolites compared to control cells, but this effect was reduced at higher concentrations (≥ 1.0 μM). Since the data of BBB permeability suggest that 4.0% (0.5 h) of EGCG can pass through blood to brain parenchyma, it may be possible to speculate how much EGCG is needed in the blood for ~0.05 µM EGCG to reach the brain [43,60]. The plasma concentration of EGCG in humans is 0.02 μM after drinking black tea containing 16.74 mg of EGCG [51]. After a few hours of circulation of blood containing 0.02 μM EGCG, its accumulation is ~0.05 µM in the brain. Although EGCG reaches in only trace amounts after 8 h or more of the EGCG intake, EGC-M5, a metabolite of EGCG, can be found in the blood. Whereas the levels of EGCG metabolites such as EGC-M5 and its conjugates in blood have not been determined, they are thought to be circulating in the blood for several hours. Since the BBB permeability of EGC-M5 is slightly higher than that of EGCG and the bioavailability of catechins is reported to be 39% in 24 h [52] and 62% in 48 h [54], EGC-M5 transferred from blood into the brain may also have a role in neuritogenesis. It is necessary to further investigate whether EGCG and its metabolites reach concentrations that cause neuritogenesis in vivo after consuming several cups of green tea per day in humans.

## 3. Bioactivity of Catechin Ring-Fission Metabolites

Catechin metabolites show several biological activities, including anti-oxidative, anti-inflammatory, anti-cancer, immunomodulatory, anti-thrombotic, and blood pressure-lowering activities (Table 3).

Hara-Terawaki et al. evaluated anti-cancer effects of catechin metabolites against human cervical cancer cells (HeLa cells) [62]. The authors screened the inhibitory activities of 11 kinds of metabolites (EGC-M1-M11) produced from EGCG by intestinal microbiota on proliferation of HeLa cells. Among the 11 metabolites, EGC-M1, EGC-M6, and EGC-M10 inhibited the proliferation of HeLa cells at a final concentration of 50 μg/mL [62]. Another study by Takagaki et al. investigated the anti-oxidative activity of catechin metabolites by flow injection analysis coupled to an on-line antioxidant detection system with the 2, 20-azinobis (3-ethylbenzothiazoline-6-sulfonic acid) radical cation. The radical scavenging abilities of EGCG metabolites, such as EGC-M4, EGC-M5, EGC-M9, EGC-M10, and EGC-M11, as well as 5-(3, 4 dihydroxyphenyl)-γ-valerolactone, and 5-(3-hydroxyphenyl)-γ-valerolactone), which are ring-fission metabolites produced from EC or ECG, were found to be stronger than those of parental catechins [63]. Two ring-fission metabolites of tea catechins were tested for their anti-cancer and anti-inflammatory activities against a panel of immortalized and malignant human cell lines [64]. EGC-M7 had significantly strong inhibitory activity at 15–73 µM than 5-(3,4-dihydroxyphenyl)-γ-valerolactone at 50 µM against human colon cancer cells (HT-29 and HCT-116), human esophageal squamous cell carcinoma (KYSE150), human normal immortalized intestinal cells (INT-407), and rat intestinal epithelial cells (IEC-6). EGC-M7 also showed anti-inflammatory activity at 20 µM by inhibiting nitric oxide production (50%) in lipopolysaccharide (LPS)-stimulated murine macrophage (RAW264.7) cells [64]. The anti-oxidant activity of a ring-fission metabolite 5-(3,4-dihydroxyphenyl)-γ-valerolactone from (−)-epicatechin was described by Unno et al. [65]. In another study, EGC-M5 was found to have immunomodulatory activity by enhancing the activity of CD4^+^ T cells and the cytotoxic activity of natural killer cells in BALB/c mice [66]. EGCG microbial metabolites were found to have blood pressure lowering activity in rats. A single oral intake of EGCG metabolites, EGC-M5 and EGC-M7, was examined to observe systolic blood pressure (SBP) using spontaneously hypertensive rats. There was a significant decrease in SBP 2 h after administration (150 mg/kg) of EGC-M7 and 4 h after administration (200 mg/kg) of EGC-M5, compared to the control group [67]. More recently, EGCG microbial metabolites were found to have antidiabetic effects in vitro and in vivo [41]. Glucose uptake ability of EGCG metabolites was measured with differentiated rat L6 myoblast cells by using 2-deoxyglucose. The treatment with EGC-M5, EGC-M6, EGC-M7, and EGC-M11 at 3 μM for 15 min significantly increased glucose uptake by 164.2%, 165.2%, 167.6%, and 146.3%, respectively, compared to control cells [41]. Moreover, oral administration of EGC-M5 at 32 mg/kg of body weight significantly suppressed postprandial hyperglycemia at 15 min (150.5 ± 13.6 mg/dL) and 30 min (108.5 ± 17.2 mg/dL) after oral glucose loading, compared to the saline control group [41].

The above studies indicate an important contribution of intestinal microflora-derived ring fission metabolites of catechins on protection against various diseases, including neurodegenerative diseases.

## 4. Conclusions and Future Expectation

Several studies including animal, human, and cell cultures support the potential neuroprotective activities of green tea catechins against neurological disorders. Very recently, EGCG was found to be safe and potential in improving cognition using both preclinical (mice) and clinical (human) studies [68]. The concentrations of EGCG, which is the main and the most active component among catechins, are very low in human and rat plasma and EGCG disappears within several hours from systemic circulation (<8 h) due to fast and extensive metabolism (methylation, glucuronidation, and sulfation) and microbial metabolism and degradation, resulting in the formation of various microbial ring-fission metabolites, which are detectable (>8 h) in the plasma and urine [30,31,33]. These microbial ring-fission metabolites show much higher bioavailability [52,55]. Intact EGCG and its metabolites reached the brain parenchyma through the BBB and induced neuritogenesis at a low concentration (0.05 µM) [43,60].

Based on our and other findings, we propose a possible action of EGCG and its metabolites in the brain as follows. When humans drink green tea, intact EGCG at a very low micromolar level reaches the brain parenchyma through the BBB and may induce neurite outgrowth, and after EGCG disappears, metabolized EGCG may promote neurite outgrowth, resulting in the prevention of cognitive dysfunction [43,60]. On the other hand, EGCG and its metabolites that reached the brain may reduce oxidative damage, since the levels of lipid peroxidation were significantly reduced in the brain of senescence-accelerated mouse prone 10 (SAMP10) that ingested EGCG [60]. In addition, EGCG metabolites have anti-oxidant activity [63,65]. Thus, microbial ring-fission metabolites may play an important role in suppressing brain dysfunction. However, differences in intestinal microbiota may have great importance on the variability of metabolites as well as the absorption rate among humans [52,53,54,69]. To date, there are no findings on the neuroprotective action of microbial ring-fission metabolites of EGCG in vivo. It is becoming epidemiologically clear that intake of green tea suppresses cognitive decline [11,70,71]. In the future it will be necessary to examine not only the relationship between green tea intake and brain function but also the relationship between brain function and the concentrations of EGCG and its metabolites in the blood.

## Figures and Tables

**Table 1:** Microbial ring-fission metabolites of EGCG in rat.

EGCG Metabolites (Microbial Ring-Fission)	Abbreviation
1-(3,4,5-trihydroxyphenyl)-3-(2,4,6-trihydroxyphenyl)-propan-2-ol	(EGC-M1)
4-dehydroxylated epigallocatechin	(EGC-M2)
1-(3,5-dihydroxyphenyl)-3-(2,4,6-trihydroxyphenyl)-propan-2-ol	(EGC-M3)
4-hydroxy-5-(3,5-dihydroxyphenyl) valeric acid	(EGC-M4)
5-(3,5-dihydroxyphenyl)-γ-valerolactone	(EGC-M5)
4-hydroxy-5-(3,4,5-trihydroxyphenyl) valeric acid	(EGC-M6)
5-(3,4,5-trihydroxyphenyl)-γ-valerolactone	(EGC-M7)
3-(3,5-dihydroxyphenyl) propionic acid	(EGC-M8)
5-(3,5-dihydroxyphenyl) valeric acid	(EGC-M9)
5-(3,4,5-trihydroxyphenyl) valeric acid	(EGC-M10)
5-(3-hydroxyphenyl) valeric acid	(EGC-M11)

Adapted from Takagaki et al. [41].

**Table 2:** BBB permeability of EGCG metabolites.

Sample	Permeability Coefficient (10^−6^cm s^−1^)	BBB Permeability (%) (30 min)
EGCG	13.45 ± 0.57	4.00 ± 0.17
EGC	16.70 ± 1.86	4.96 ± 0.55
GA	31.73 ± 3.39	9.42 ± 1.01
EGC-M5	17.99 ± 0.79	5.34 ± 0.23
EGC-M5-GlcUA	12.53 ± 0.02	3.72 ± 0.01
EGC-M5-Sul	14.61 ± 1.35	4.34 ± 0.40
PG	13.79 ± 1.62	4.10 ± 0.48
PG-GlcUA	9.28 ± 1.41	2.76 ± 0.42

Data are expressed as the mean ± SEM (*n* = 3) [43]. (Data of Ref. 43 are modified).

**Table 3:** Bioactivity of catechin metabolites.

Catechin Metabolites	Bioactivity	Reference
5-(3,4-dihydroxyphenyl)-γ-valerolactone	Anti-oxidative	[63]
5-(3,4-dihydroxyphenyl)-γ-valerolactone	Anti-oxidative	[65]
5-(3-hydroxyphenyl)-γ-valerolactone	Anti-oxidative	[63]
(EGC-M1)	Anti-cancer	[62]
(EGC-M4)	Anti-oxidative	[63]
(EGC-M5)	Antidiabetic effect	[41]
(EGC-M5)	Neuritogenic activity	[43]
(EGC-M5)	Blood–brain barrier penetrating activity	[43]
(EGC-M5)	Anti-oxidative	[63]
(EGC-M5)	Immunomodulatory activity	[66]
(EGC-M5)	Blood pressure lowering activity	[67]
(EGC-M6)	Antidiabetic effect	[41]
(EGC-M6)	Anti-cancer	[62]
(EGC-M7)	Antidiabetic effect	[41]
(EGC-M7)	Anti-cancer	[64]
(EGC-M7)	Anti-inflammatory	[64]
(EGC-M7)	Blood pressure lowering activity	[67]
(EGC-M9)	Anti-oxidative	[63]
(EGC-M10)	Anti-oxidative	[63]
(EGC-M10)	Anti-cancer	[62]
(EGC-M11)	Antidiabetic effect	[41]
(EGC-M11)	Anti-oxidative	[63]
